# A Bagworm-Inspired Robot That Acquires Its Exterior from External Environments

**DOI:** 10.3390/biomimetics10040252

**Published:** 2025-04-20

**Authors:** Noriko Ishida, Mitsuharu Matsumoto

**Affiliations:** Graduate School of Informatics and Engineering, University of Electro-Communications, 1-5-1, Chofugaoka, Chofu-shi, Tokyo 182-8585, Japan; i2110057@gl.cc.uec.ac.jp

**Keywords:** bagworm-inspired robot, robot skin, external environment

## Abstract

In this research, we propose a bagworm-inspired robot that can acquire its exterior by incorporating various objects from the surrounding environment into its skin. This study was inspired by the bagworm, the larva of the giant bagworm moth, which wraps itself around straw and other materials to use as a nest. When the robot is active outdoors, it is surrounded by natural materials such as sand, fallen leaves, and pieces of wood, and can change its skin by attaching or detaching these materials as needed. In the previous study, the authors developed a camouflage robot that assimilates with the outside world by incorporating natural environmental sand. In this study, by using a water-soluble adhesive as the adhesive material, it is possible to take in a larger number of external substances than before. We also conducted experiments with natural materials, including leaves and pebbles, and confirmed that the robot could pick them up. We expect that by developing these functions, robots will not only have camouflage capabilities but also the ability to reinforce their own skin like bagworms.

## 1. Introduction

Recently, natural disasters have been occurring frequently, increasing the demand for disaster rescue robots that can operate in disaster areas and assist with search and rescue efforts [1,2,3]. In these disaster scenarios, various types of robots have been developed to perform well in a variety of environments. Crawler mechanisms [4,5,6] and snake-like robots [7,8,9] are examples.

However, in disaster areas, there are constant risks of secondary disasters due to soil loosening and aftershocks, which necessitate countermeasures to address external damage leading to robot malfunctions or breakage.

Approaches to dealing with such external damage include self-repair [10,11,12], reinforcement using additional materials [13,14,15], and coating technologies that either prevent or minimize damage through defensive coatings [16].

In disaster areas, there are many situations where human intervention becomes difficult when a robot is damaged or broken. Hence, this study focuses on self-repair mechanisms. Although there have been many studies on robot self-repair, most of them rely on utilizing the material properties inherent to the robot, such as phase transitions [17,18,19,20] or chemical changes [21], to self-repair using the materials that constitute the robot itself. Therefore, when materials are lost or insufficient, applying these traditional approaches becomes difficult.

In recent years, many robots have been developed that mimic the mechanisms, movements, and structures of living organisms in nature. By imitating biological characteristics and incorporating efficient and adaptive movements or structures, the development of robots with a superior performance is anticipated. Examples are snake-like robots capable of flexible movements in confined spaces [22,23,24,25] and butterfly-like robots that mimic the flight mechanism of butterflies to achieve quiet and fast aerial movement [26,27,28]. Research into mathematical models of biology is also progressing [29].

Biomimetics is being widely studied not only in the field of robotics but also in mechanical engineering in general, with inspiration being drawn from animals on land, sea, and air [30] and being applied to a variety of materials and industrial products [31].

The bagworm, a larva of Eumeta japonica, wears the nest it produces and uses it as a kind of exterior [32]. The bagworm uses fiber from its surroundings during larval stages to create a nest that it wears around itself. Birds live in their own nests but do not wear them like bagworms. For this reason, a bird’s nest protects the bird from the cold and heat of the outside world, but it does not act as armor to protect it from predators. In contrast, bagworms cover themselves with their webs, which not only protect them from the cold and heat outside but also act as armor to protect them from predators. Even if the nest is broken, the bagworm reassembles the nest, or exterior, by collecting materials from the outside. This not only allows the nest to camouflage itself from the outside environment but also increases its own strength. Inspired by this method, Nozawa and colleagues developed a camouflage robot that adheres external objects to its skin and assimilates with the surrounding environment [33]. However, this research focused on the robot’s camouflage function, and the materials collected from the outside were limited to small particles such as sand.

Our approach may also be relevant to research on self-organization. Self-assembly is a method of assembling multiple modules by itself [34,35], rather than pick-and-place [36,37] or other methods. Modular robots are also related to automotive self-assembly [38]. In most research on self-organization, the components of the robot are deformed to form the desired shape. In modular robots, multiple robots are combined with each other to create a shape. Unlike conventional self-organization research, we aim to utilize materials that are naturally present in the external environment as the building blocks of the robot.

If a robot can create a new exterior from materials available in its environment, it can not only respond to losses and damages caused by external factors but also enhance its own structure on site. This technology holds great potential for use in disaster rescue robots that support disaster sites and search and rescue operations.

This research aims to develop a robot that can absorb larger materials from the surrounding environment and has skin that can be removed when it is no longer needed. Specifically, the robot will absorb fallen leaves, sand, wood chips, and other materials present in the environment onto its skin as it moves through the surroundings.

## 2. Design Plan

### 2.1. Fundamental Plan

**Figure 1:**
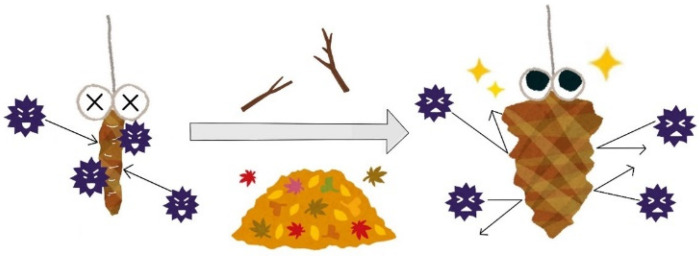
Conceptual diagram of bagworm nest.

Here we explain the basic plan of the robot. Figure 1 shows a conceptual diagram of the bagworm nest. The bagworm, when without its bag (exterior), is vulnerable to external stimuli and dangerous. Therefore, it collects external materials and uses them as a type of exterior. In this study, we focus on this approach taken by bagworms. In the proposed approach, the robot incorporates surrounding materials, treating external materials as part of its own structure.

In this study, we refer to the approach of the bagworm, where external materials are treated as part of its own structure, and the robot incorporates materials from its environment to enhance its strength. We also consider mechanisms for removing collected materials from the robot when they are no longer needed.

Regarding external materials, we suppose natural environmental elements such as sand, fallen leaves, and wood chips. In previous research, water was used as an adhesive for incorporating external materials, but it was only effective for light substances such as sand. This study examines the incorporation of larger and heavier substances such as small stones, fallen leaves, and wood chips. To solve this issue, adhesive requirements were determined, and preliminary experiments were conducted, which will be discussed later.

**Figure 2:**
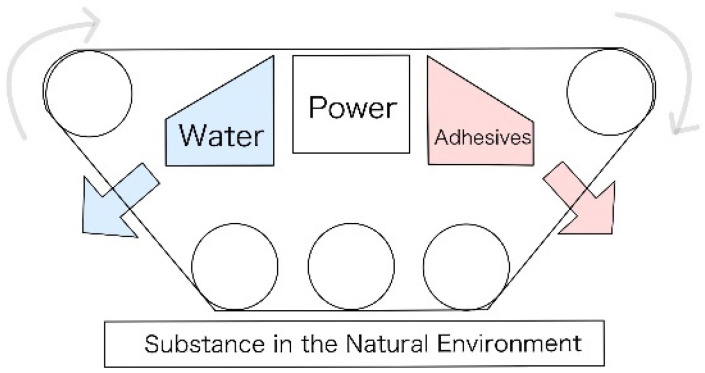
Basic concept of robot design.

Figure 2 shows an overview of the robot. As illustrated in Figure 2, a robot with an endless track mechanism was adopted for the basic part of the robot. During implementation, a servo motor will be used at the front of the robot to extrude adhesive and incorporate it into the robot’s skin. This will allow external materials to adhere to the skin. Additionally, a mechanism for releasing peeling liquid will be mounted on the rear. If the incorporated materials are no longer needed, the peeling liquid will be released to remove the substances. These two implementations will enable the robot to attach and detach external materials as necessary.

### 2.2. Detailed Plan

The detailed plan includes the examination and determination of the robot’s structure, skin requirements, adhesive requirements, and the mechanism for extruding liquids.

First, as a robot capable of attaching and detaching its exterior, the following two points were considered:

- The robot must be able to reach its destination even on difficult terrain;
- The robot must be able to interact with the external environment.

Based on these requirements, a crawler mechanism was selected for the robot. Moreover, by sewing the skin to the endless track of the robot, the contact area between the skin and the ground will change as the robot moves. This design ensures that the robot comes into contact with the ground at regular intervals during its operation.

We also set the following requirements for the adhesive:

1. The adhesive must be strong enough to incorporate external materials;
2. The adhesive should have low viscosity for ease of handling;
3. The adhesive should not negatively impact the natural environment.

We tried various adhesives, including water, instant adhesives, liquid adhesives, and water-soluble adhesives. Table 1 shows the adhesive requirements and comparison. Water has low adhesive strength and viscosity. Instant adhesives have strong adhesion but are difficult to handle due to their high viscosity. Liquid adhesives have low viscosity and strong adhesion, but they may have environmental impacts. Among these options, water-soluble adhesives were chosen because they meet all the requirements: they have strong adhesion, low viscosity, and are easy to handle, as well as being environmentally friendly since they do not use organic solvents. For the peeling liquid, water was selected.

The liquid extrusion mechanism will be designed to release liquid only when necessary and to hold the liquid otherwise. Details of the extrusion mechanism will be described in the next section.

## 3. Robot Design

### 3.1. Robot Structure

**Figure 3:**
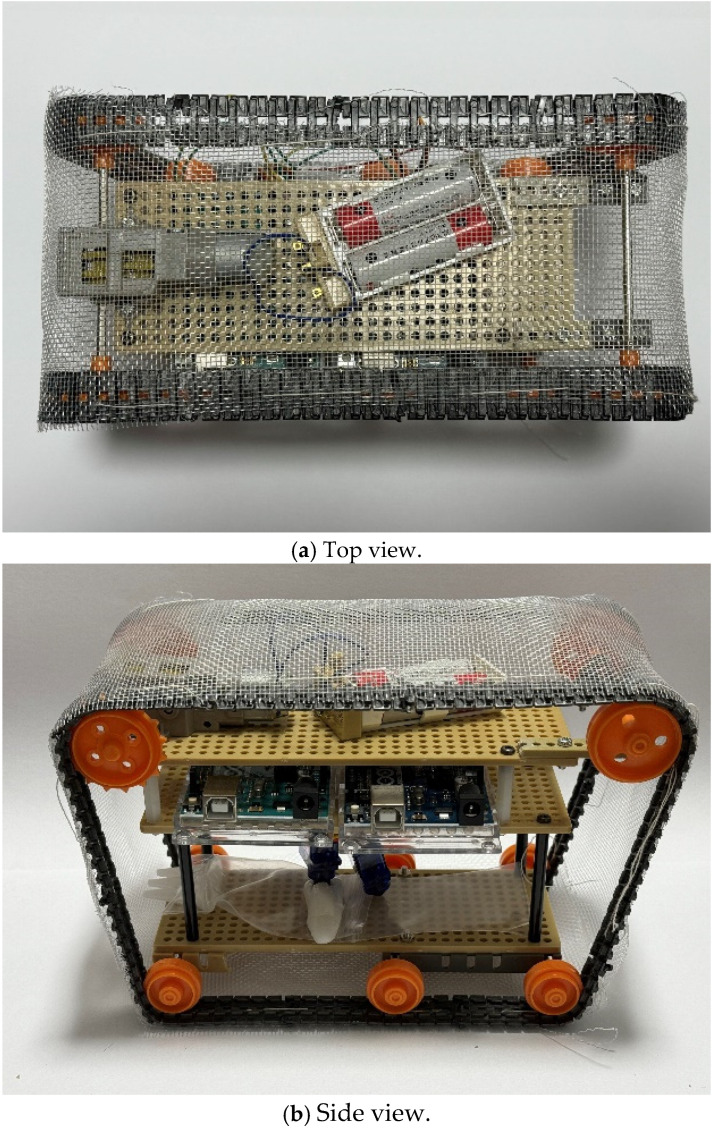
Robot appearance (skin included). (**a**) The top view of the robot (**b**) The side view of the robot.

**Figure 4:**
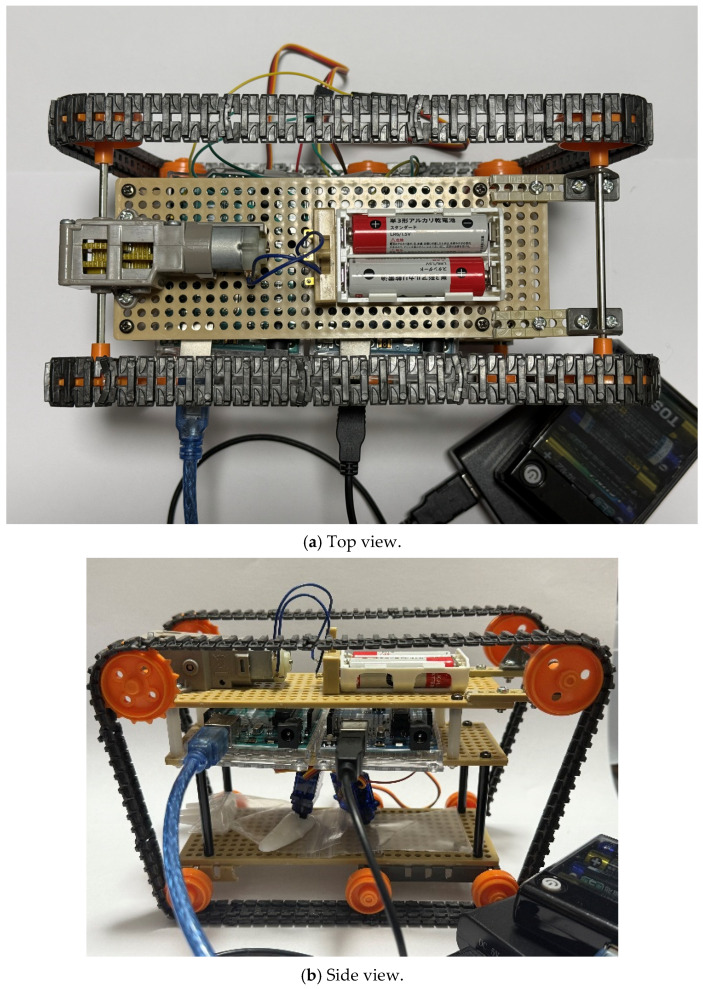
Robot appearance (skeleton). (**a**) The top view of the robot (**b**) The side view of the robot.

**Figure 5:**
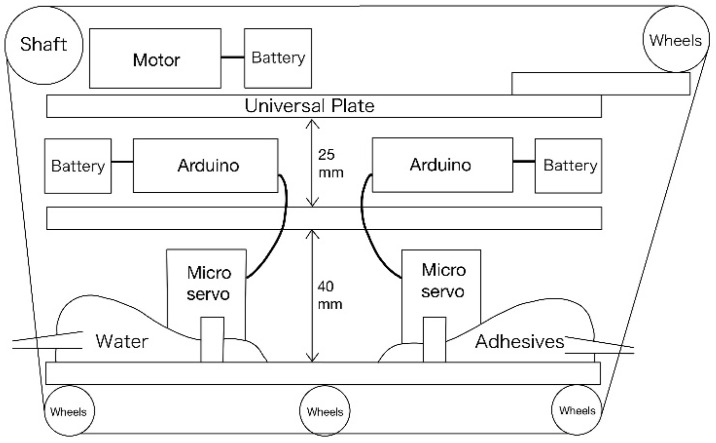
Parts layout diagram of the robot (side view).

**Figure 6:**
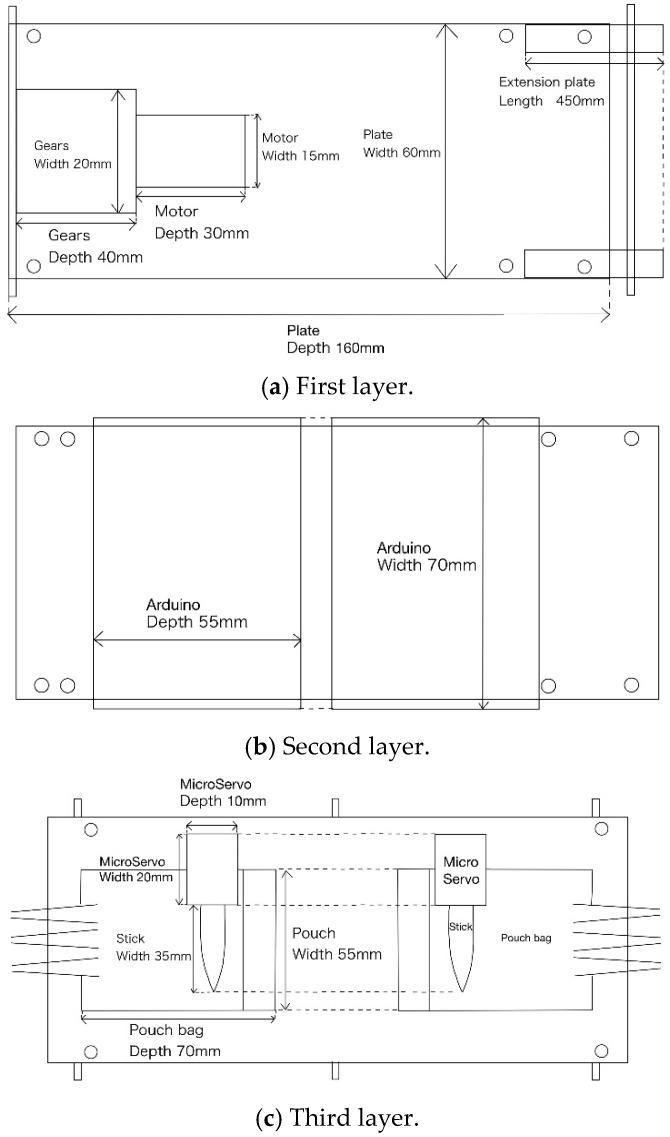
Parts layout diagram of the robot (top view). (**a**) The layout of the first layer (**b**) The layout of the second layer (**c**) The layout of the third layer.

This section describes the design of the robot’s body and its internal structure. Figure 3 shows the top and side views of the robot we created, and Figure 4 shows the top and side views of the robot without the skin material. The skin covering the robot’s surface is sewn onto the robot’s endless tracks. The rotation of the skin is controlled by a servo motor via gears, which allows the robot to move forward and backward. The mechanism for extruding liquid into the skin to adhere and detach external materials will be discussed later. This mechanism is positioned at the center of the robot. Figure 5 shows the component layout when viewed from the side, while Figure 6a–c show the component layout from above for layers 1 through 3.

Next, we will describe the details of the parts used in the robot. The basic structure of the robot uses the Tamiya Endless Track Vehicle Kit No. 70108 (Tamiya Inc., Shizuoka, Japan). The original robot is 171 mm long, 105 mm wide, and 56 mm high. For robot control, two AA batteries and a battery box with a reverse switch were used for manual control. The original robot is 171 mm long, 105 mm wide, and 56 mm high. The weight of the developed robot is 399 g. The contact area with the floor is 180 cm^2^. So, the pressure on the ground is thought to be around 2.2 g/cm^2^ on average. The robot’s speed is about 6 cm/s. As for the adhesive, considering appropriate adhesion and peel strength as well as environmental impact, we used the water-soluble adhesive ECOSTICK-1816B (by Intercom).

### 3.2. Skin Design

This section describes the skin used for the robot. In this research, external materials such as natural environmental sand, fallen leaves, and wood chips are adhered to the skin using water-soluble adhesives and can be detached when necessary by using water. Therefore, we set the following two requirements for the robot skin:

- The peeling liquid (water) should not remain on the skin after removal;
- The texture should be sufficiently rough to allow easy removal of unwanted material.

The materials considered for the skin were hemp, cotton, nylon mesh #40, and nylon mesh #18. We are conducting qualitative experiments to select the skin of the robot. Through the experiments, it was found that cotton and linen have a high ability to adhere adhesive to themselves, and when trying to apply the adhesive to the outside, much of it ends up sticking to the fabric itself. On the other hand, when nylon mesh was used, the adhesive adhered to the nylon but could be easily peeled off as needed, making it suitable for use.

Comparing nylon mesh #40 and nylon mesh #18, nylon mesh #40 has an opening of 526 μm while nylon mesh #18 has an opening of 1242 μm. When comparing nylon mesh #40 with nylon mesh #18, nylon mesh #40 is more than twice as fine. For this reason, nylon mesh #40 had a tendency to have poor adhesive flow and become clogged. Based on these experiments, we chose nylon mesh #18.

Based on preliminary experiments, nylon mesh #18 was selected because it satisfied all the necessary criteria.

### 3.3. Mechanism for Pushing Out Liquid

This section describes the design for the mechanism that pushes out liquid. In this research, we are considering extruding liquid from the robot body to adhere and detach external materials on the skin when necessary. Two methods for extruding liquid were considered:

- Using a motor to extrude the liquid through a three-hole dispenser;
- Installing a platform to release the liquid like a dam.

**Figure 7:**
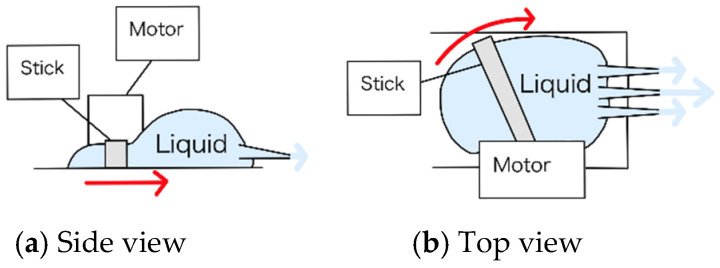

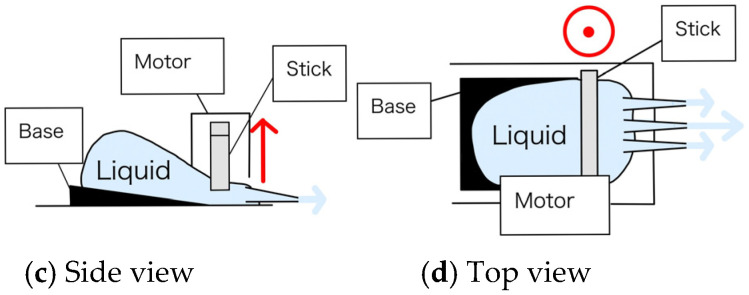
Schematic diagram of liquid extrusion mechanism. The red arrow represents the movement of the stick. A red circle with a dot in the center indicates an arrow pointing from the back to the front. (**a**) The side view of the first prototype (**b**) The top view of the first prototype (**c**) The side view of the second prototype (**d**) The top view of the second prototype.

Figure 7 shows the overview of the liquid extrusion mechanism. The first design for the liquid extrusion mechanism is shown in Figure 7a (side view) and Figure 7b (top view). The liquids considered for this process are the adhesive and water. The adhesive requires moderate viscosity and needs to be supplied over a wide area, so a design with a three-hole dispenser was selected. In this design, a rod attached to a servo motor rotates within a pouch of adhesive, pushing it out.

Although a similar mechanism was considered for water, there was concern that water, with its low viscosity, might leak out unintentionally. Therefore, for the water extrusion mechanism, a platform was designed (Figure 7c,d) where gravity could assist in the flow of water. The servo motor is used in the same way as for the adhesive, where the rod moves upward to release the water, similar to a dam design.

Through experiments, it was determined that water would not leak due to the thickness of the three-hole dispenser. Therefore, for both adhesive and water, the first mechanism will be used to extrude the liquid.

**Figure 8:**
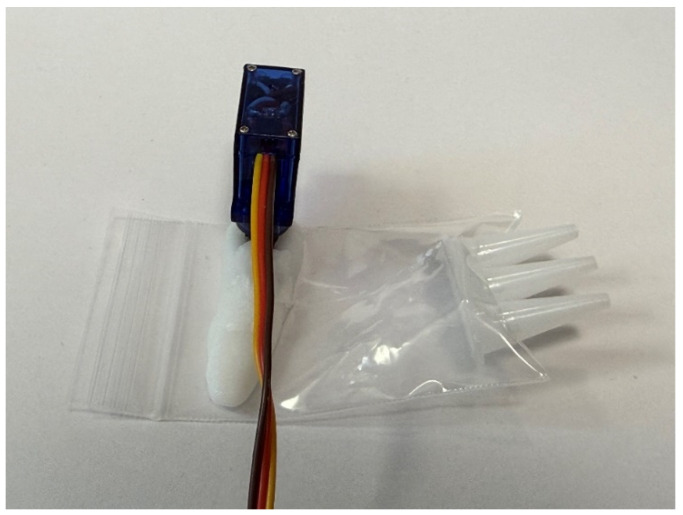
Liquid extrusion mechanism.

**Figure 9:**
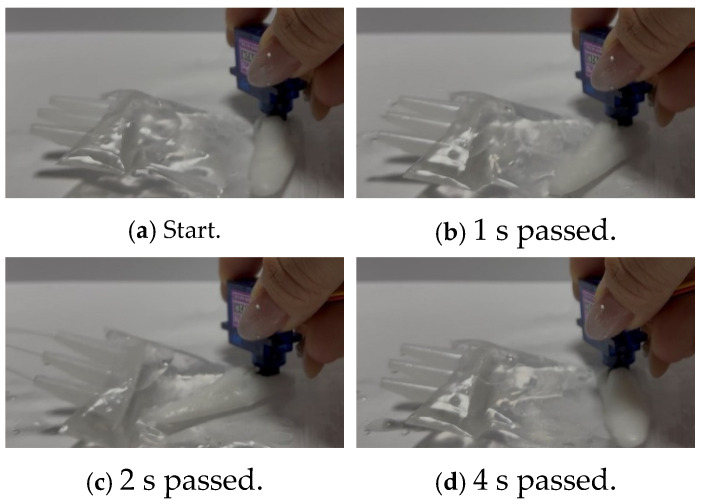
Liquid extrusion mechanism in action. (**a**) The photograph of the initial state (**b**) The photograph taken 1 second after the stick moved (**c**) The photograph taken 2 seconds after the stick moved (**d**) The photograph taken 4 seconds after the stick moved.

The actual liquid extrusion mechanism we built is shown in Figure 8. Additionally, Figure 9a–d show the process of liquid being extruded, from 0 s to 3 s after extrusion.

## 4. Design of Outside Environment

### 4.1. Environment Setup

**Figure 10:**
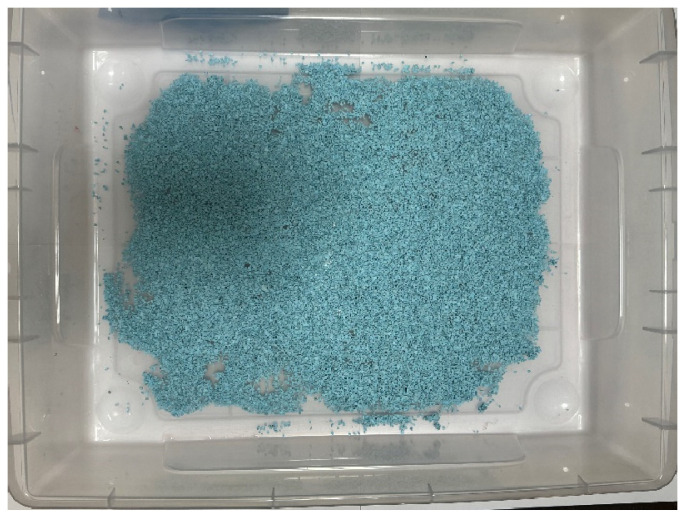
External environment laid with Color Zeolite.

**Figure 11:**
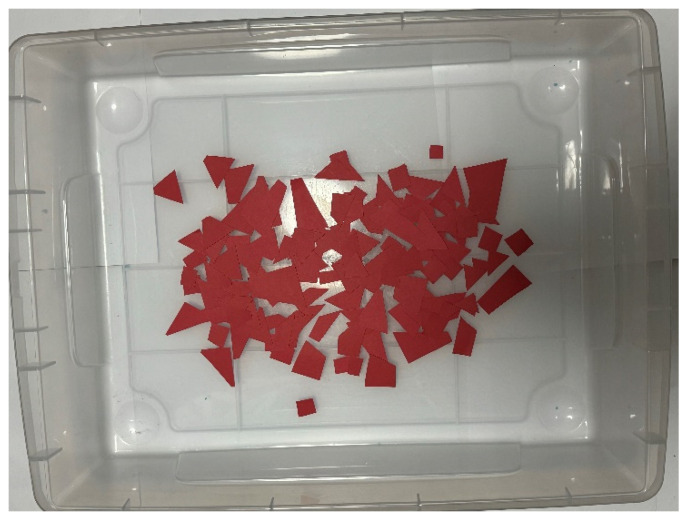
External environment laid with red papers.

**Figure 12:**
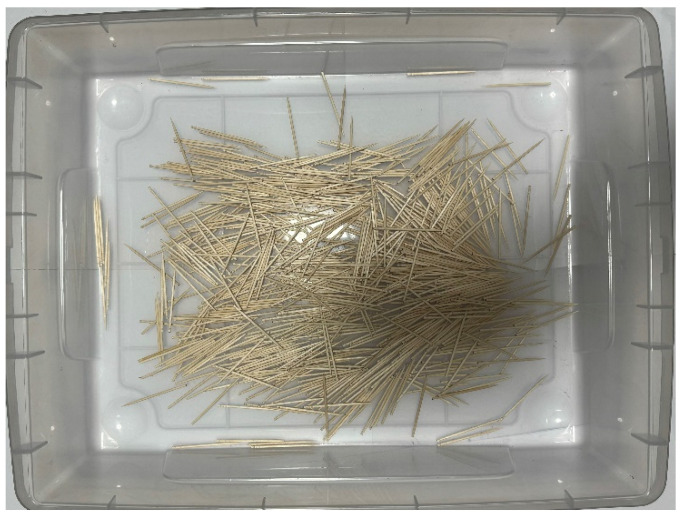
External environment laid with toothpicks.

This section discusses the setup of the external environment in which the robot operates. In the future, we would like to use the robots we develop outdoors, such as in natural environments or at disaster sites. In this experiment, we aim to measure the robot’s collection capabilities in various external environments by preparing multiple objects in the external environment and conducting the experiment. In this research, natural materials such as sand, small stones, fallen leaves, and wood chips are considered external substances. As substances similar to the assumed environment, materials such as colored zeolite, paper scraps, and toothpicks were prepared for the external environment. Figure 10, Figure 11 and Figure 12 show how these materials are arranged in the environment. The box dimensions are 54 cm length × 40.5 cm width × 13.5 cm height. By arranging the materials in the environment, we make changes visually easier to perceive.

Zeolite is a porous material with holes around a few nanometers in diameter, used as a buffer or adsorbent. In the experiment, Color Zeolite (DAISO), color paper cut into pieces from 1 cm to 5 cm, and toothpicks were placed in the environment. The size of the colored zeolite was 1 mm minimum and 4 mm maximum. The length toothpicks were about 6.5 cm.

### 4.2. Experimental Method

This section describes the verification experiments conducted to evaluate our robot’s ability to adhere and peel the exterior from external environments. In these verification experiments, the robot was controlled using two AA batteries and a battery box with a reverse switch. The liquid extrusion mechanism was controlled by a servo motor connected to an Arduino on a personal computer. The servo motor used was the Tower Pro SG92R Micro Servo (Tower Pro Pte Ltd, Singapore). The rod used to extrude the liquid was made of plastic with appropriate viscosity.

As for the adhesive, the water-soluble adhesive ECOSTICK-1816B (manufactured by Intercom, Florence, Italy) was used, considering factors such as proper adhesion and peeling force and environmental impact. It is a room temperature water-based polyurethane adhesive and a completely water-soluble adhesive that does not use organic solvents. It is certified in accordance with the EU REACH regulation. The material for the skin was nylon mesh #18.

The focus of this experiment was to observe the adhesion of external substances and their subsequent behavior. The following two items were investigated:

1. Can external environmental substances be adhered to the skin of the robot?
2. If no longer needed, can these substances be removed from the skin?

### 4.3. Adhesion and Peeling Experiment

The adhesion and peeling experiments were carried out in the following four types of environments:

1. Adhesion and peeling on Color Zeolite;
2. Adhesion and peeling on paper;
3. Adhesion and peeling on toothpicks;
4. Adhesion and peeling on natural environmental materials (sand, fallen leaves, small stones, and wooden pieces).

These experiments aim to confirm whether the robot can acquire the exterior from the external environment and whether it can remove it when no longer needed.

#### 4.3.1. Adhesion and Peeling on Color Zeolite

In this experiment, it was confirmed that the robot could acquire the exterior by adhering to Color Zeolite and that it could be removed when no longer needed.

First, the adhesion experiment was conducted for 60 s. The robot changed its direction every 3 s and traveled around the environment 20 times to collect the external substances. The external environment used was as shown in Figure 10.

**Figure 13:**
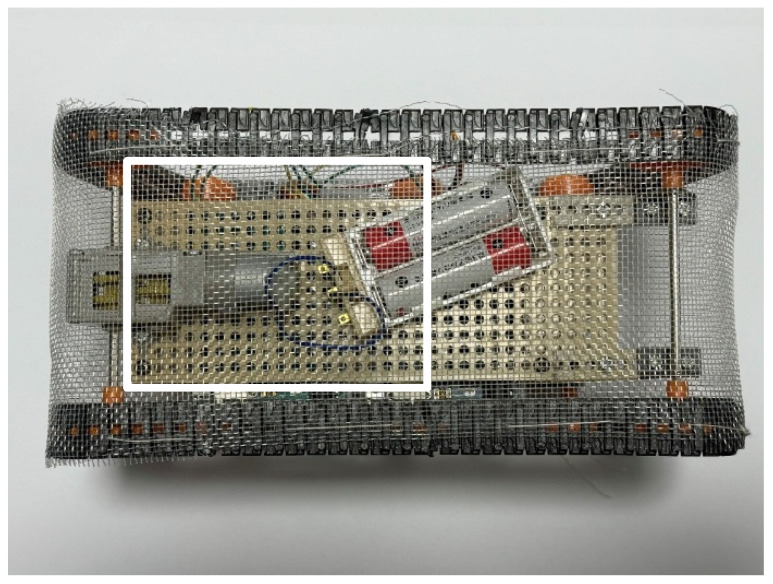
Robot appearance before adhesion experiment. The white rectangle is the area used to calculate the adhesion rate and peeling rate.

**Figure 14:**
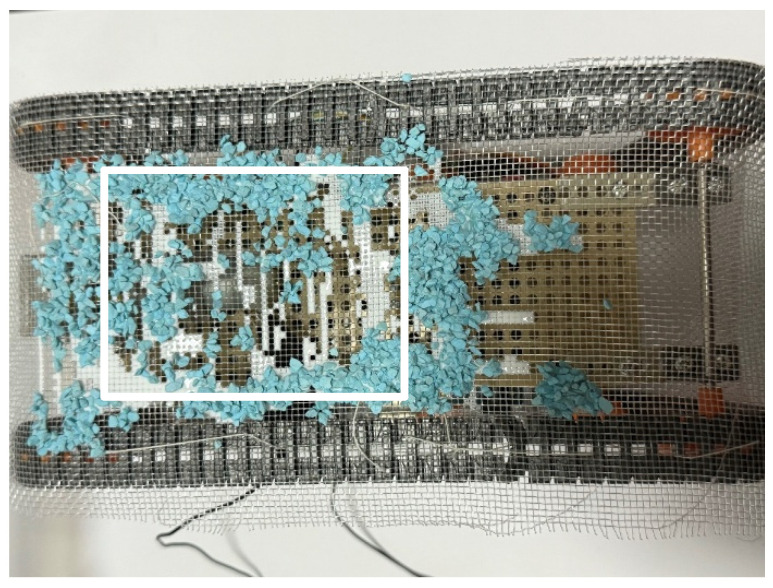
Robot appearance after adhesion experiment with robot movement for 60 s (Color Zeolite). The white rectangle is the area used to calculate the adhesion rate and peeling rate.

Figure 13 shows the robot before the experiment, and Figure 14 shows the robot after the experiment. The percentage of Color Zeolite adhered to the robot was measured. The adhesion rate *A* is defined as follows:

(1) $$A=\frac{S_{a}}{S}$$

where *S_a_* represents the area of the skin covered by the external material and *S* represents the total skin area of the robot.

We measured the adhesion rate within the white rectangle shown in Figure 13 and Figure 14. The adhesion rate after the experiment in Figure 14 was 50.5%. It is confirmed that the water-soluble adhesive allowed sufficient adhesion of Color Zeolite to the robot’s skin.

**Figure 15:**
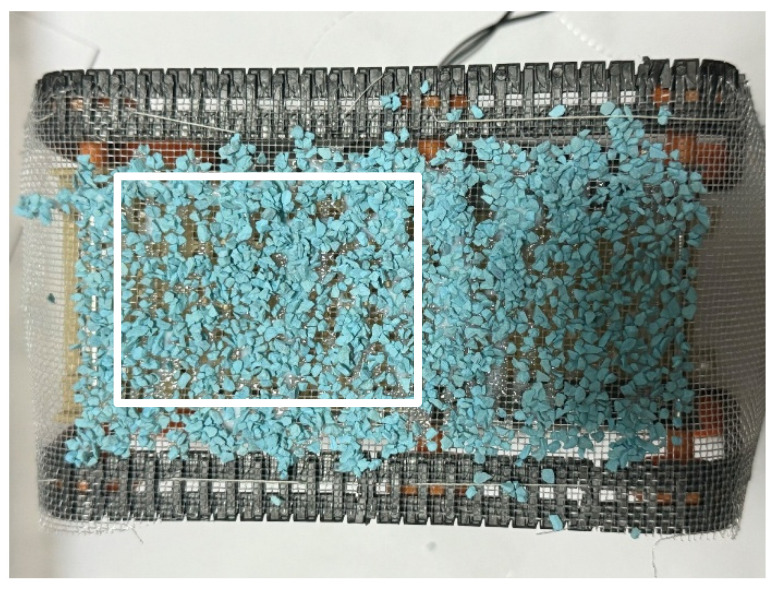
Robot appearance after adhesion experiment with robot waiting for an hour (Color Zeolite). The white rectangle is the area used to calculate the adhesion rate and peeling rate.

Next, a stationary experiment was conducted after the adhesion experiment. The robot traveled around the environment in the same manner as in the adhesion experiment and then stopped for 1 h. Figure 15 shows the robot after the experiment. The adhesion rate after this stationary period, as measured from the rectangular area in Figure 15, was 91.1%.

**Figure 16:**
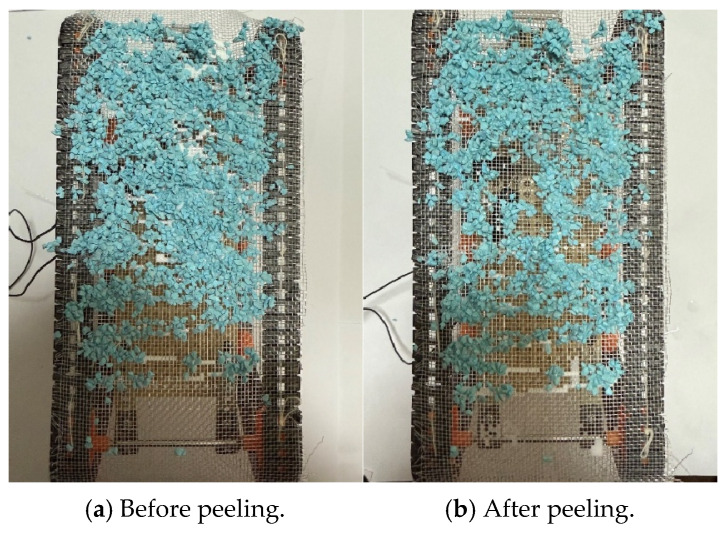
Robot appearance before and after peeling experiment when we used Color Zeolite. (**a**) The photograph before peeling (**b**) The photograph after peeling.

The peeling experiment was then performed for 60 s. External substances were adhered to the robot, and after 5 min, the robot traveled in the environment, where water was applied to peel the substances off. The mass of the Color Zeolite initially adhered to the robot was 20 g. Figure 16a,b are before and after the peeling experiment, respectively.

The peeling rate *P* was defined as follows:

(2) $$P=\frac{M_{P}}{M}$$

where *M_P_* represents the mass of the peeled external material and *M* represents the mass of the external material attached to the robot before peeling. We measured the peeling rate within the white rectangle shown in Figure 13 and Figure 15.

The peeling rate after the experiment was 33.3%. While complete peeling was not achieved, it was confirmed that some peeling was possible by using water.

#### 4.3.2. Adhesion and Peeling on Paper

In this experiment, it was confirmed that the robot could acquire the exterior by adhering to pieces of paper and that the paper could be removed when no longer needed. The experiment followed the same procedure as the adhesion and peeling experiment on Color Zeolite.

**Figure 17:**
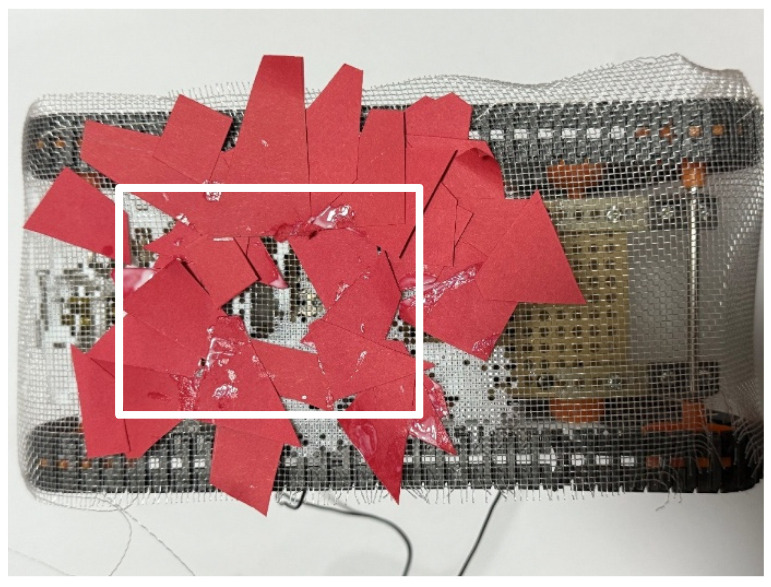
Robot appearance after adhesion experiment with robot movement for 60 s (red papers). The white rectangle is the area used to calculate the adhesion rate and peeling rate.

First, the adhesion experiment was conducted, with the external environment consisting of paper pieces ranging from 1 to 10 cm^2^. Figure 17 shows the robot after the experiment. The adhesion rate was measured from the white rectangular area in Figure 17, resulting in an adhesion rate of 88.1%.

**Figure 18:**
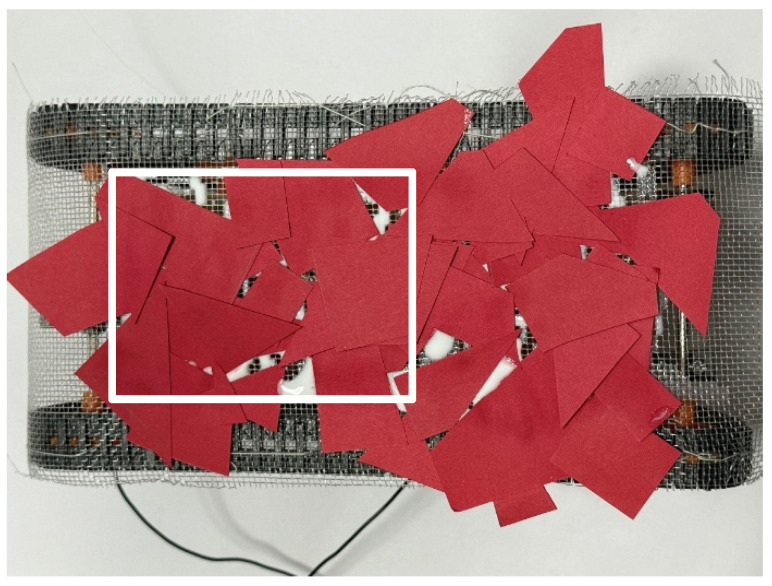
Robot appearance after adhesion experiment with robot waiting for an hour (red papers). The white rectangle is the area used to calculate the adhesion rate and peeling rate.

Next, a stationary experiment was conducted after the adhesion experiment. The robot traveled around the environment in the same manner as in the adhesion experiment and then stopped for 1 h. Figure 18 shows the robot after this experiment, with an adhesion rate of 97.8%.

**Figure 19:**
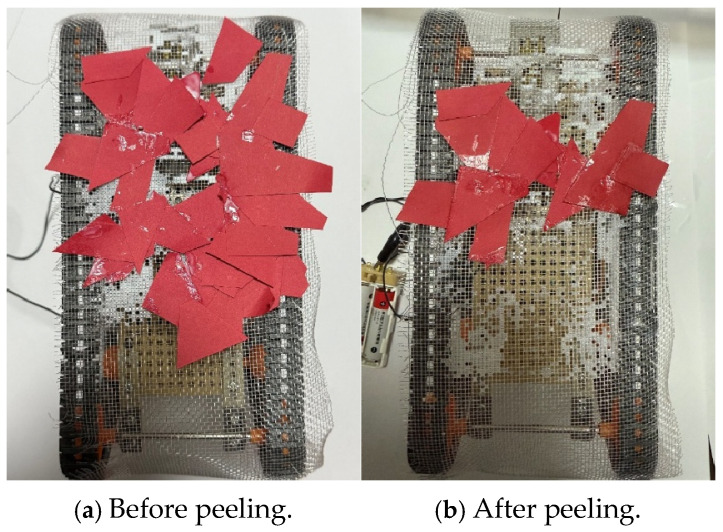
Robot appearance before and after peeling experiment when we used papers. (**a**) The photograph before peeling (**b**) The photograph after peeling.

The peeling experiment was then performed. The robot initially adhered 34 pieces of paper. Figure 19 shows the robot before and after the peeling experiment. Figure 19a shows before the experiment, and Figure 19b shows after the experiment. The peeling rate was measured by counting the number of paper pieces peeled off.

The peeling rate *P*′ was defined as follows:

(3) $$P^{\prime }=\frac{N_{P}}{N}$$

where *N_P_* represents the number of pieces of external material that were peeled off and *N* represents the number of pieces of external material that were attached to the robot before peeling. The peeling rate was 64.7%.

#### 4.3.3. Adhesion and Peeling on Toothpicks

In the experiment set in the external environment, it was confirmed that the robot could acquire the exterior by adhering to toothpicks and remove them when no longer needed. The experiment followed the same procedure as the adhesion and peeling experiments on Color Zeolite and paper.

**Figure 20:**
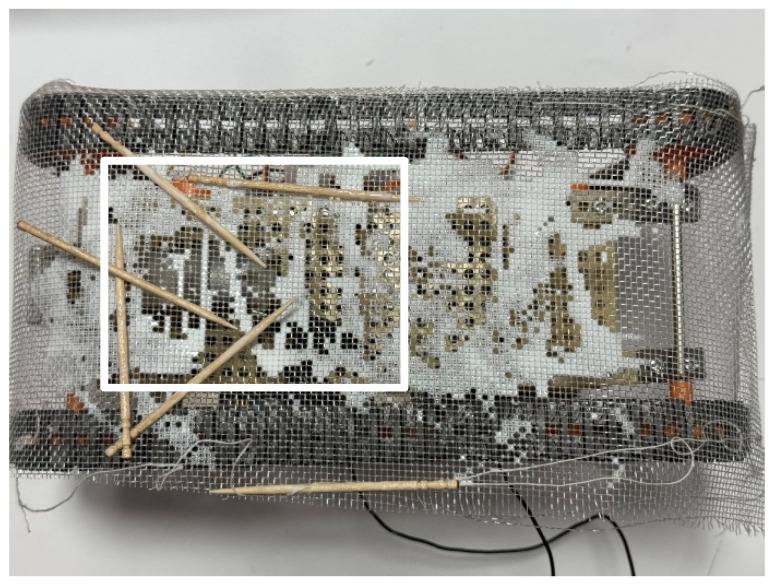
Robot appearance after adhesion experiment with robot movement for 60 s (toothpicks). The white rectangle is the area used to calculate the adhesion rate and peeling rate.

**Figure 21:**
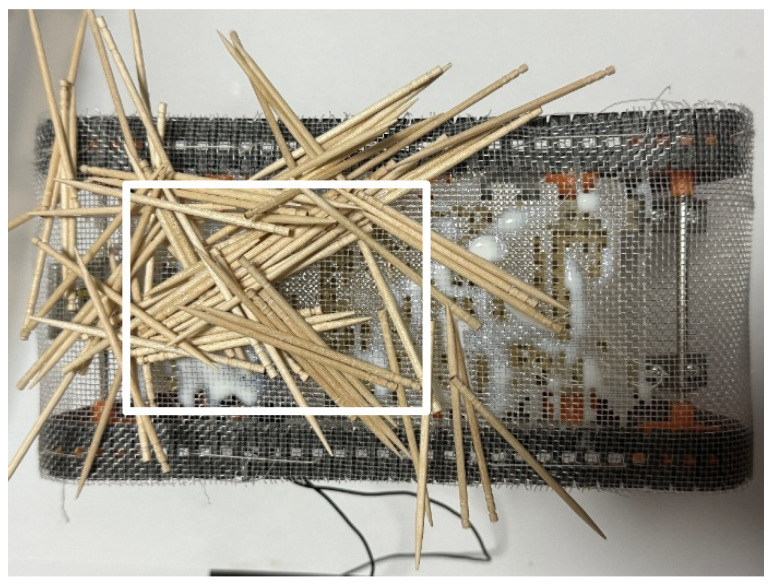
Robot appearance after adhesion experiment with robot waiting for an hour (toothpicks). The white rectangle is the area used to calculate the adhesion rate and peeling rate.

First, the adhesion experiment was conducted, with the external environment consisting of 700 toothpicks. Figure 20 shows the robot’s appearance after the experiment. The adhesion rate was measured from the rectangular area in Figure 20, and the adhesion rate was found to be 2.9%. We also conducted an experiment in which the robot was stopped for one hour after bonding. Figure 21 shows the robot after the experiment. Measurements were taken inside the rectangle in Figure 21, and the bonding rate in this experiment was found to be 41.6%. Since the adhesion was insufficient, no peeling experiment was performed.

#### 4.3.4. Adhesion and Peeling on Natural Environmental Materials

Using natural environmental materials such as sand, small stones, fallen leaves, and wooden pieces, it was confirmed that the robot could acquire the exterior from these substances and remove it when no longer needed. The experiment followed the same procedure as the adhesion and peeling experiment on Color Zeolite.

**Figure 22:**
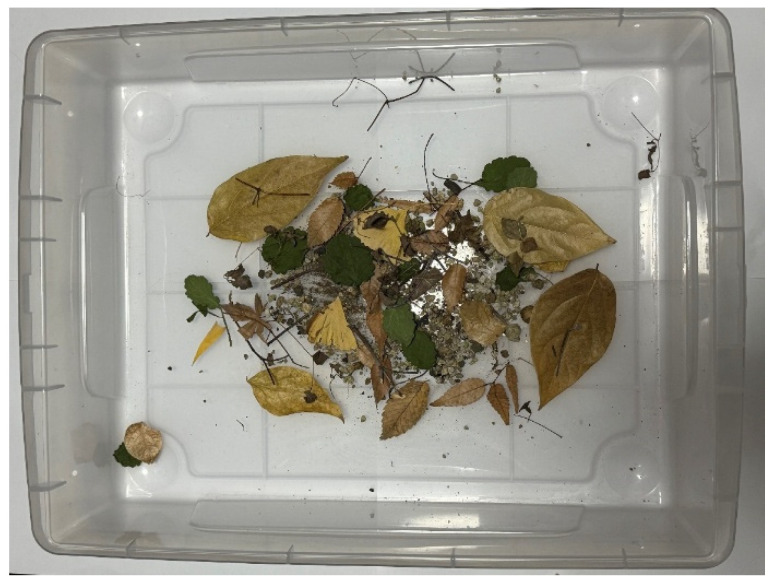
External environment with natural materials.

**Figure 23:**
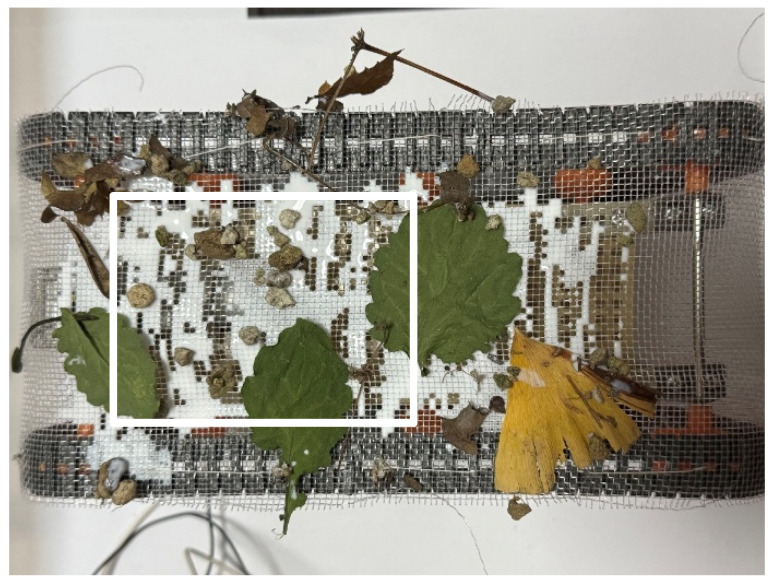
Robot appearance after adhesion experiment with robot movement for 60 s (natural materials). The white rectangle is the area used to calculate the adhesion rate and peeling rate.

**Figure 24:**
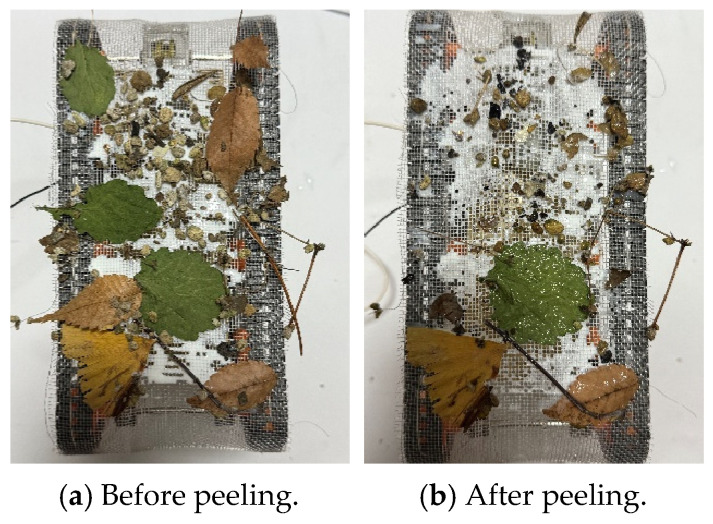
Robot appearance before and after peeling experiment.

First, the adhesion experiment was conducted with sand, small stones, fallen leaves, and wooden pieces in the external environment. Figure 22 shows the external environment used in the experiment. The external environment includes leaves up to about 10 cm in size and pebbles up to about 1 cm in size. The adhesion rate was measured as 55.9% from the robot shown in Figure 23. The maximum size of the leaves attached to the robot was about 5 cm. Next, a peeling experiment was conducted using the same materials. The peeling rate was 62.5%. Figure 24 shows the robot’s appearance before and after the peeling experiments.

### 4.4. Experiment Using Liquid Push-Out Mechanism

**Figure 25:**
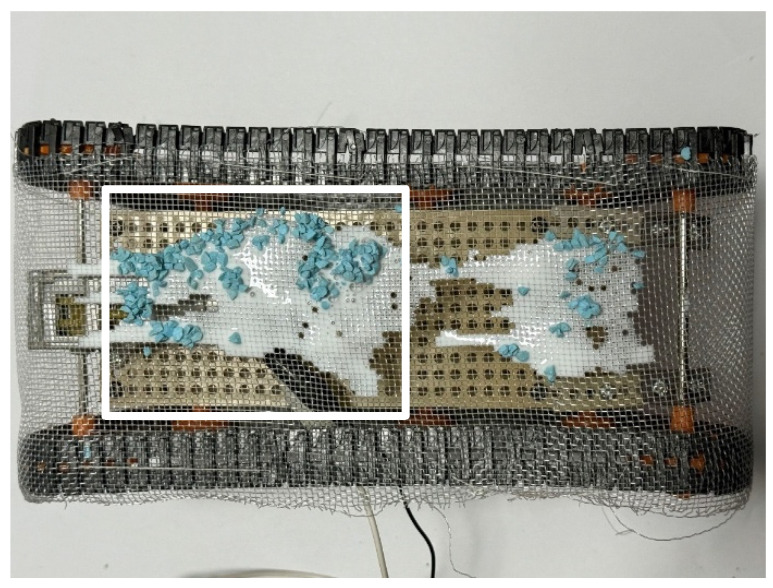
Robot appearance after experiment with liquid extrusion mechanism. The white rectangle is the area used to calculate the adhesion rate and peeling rate.

An experiment was conducted using the water extrusion mechanism added to the robot to evaluate its effectiveness. The external environment consisted of Color Zeolite and a water-soluble adhesive in an envelope, which was operated for 30 s. At the same time, the robot changed its direction every 3 s and traveled around the environment 10 times. Figure 25 shows the robot after the experiment. The adhesion rate of the Color Zeolite was measured from the rectangular area in Figure 25, resulting in an adhesion rate of 12.6%. While the result was poorer compared to previous experiments, the robot successfully adhered external substances by supplying adhesive from the liquid extrusion mechanism.

### 4.5. Discussion

Regarding adhesion, the water-soluble adhesive demonstrated sufficient adhesion to external materials such as small stones, fallen leaves, and wooden pieces. The adhesion rates ranked as follows: paper, Color Zeolite, and toothpicks. The paper adhered well because it was sewn to the robot’s skin, and the adhesive allowed several sheets to stick together, resulting in a high adhesion rate. In contrast, the toothpicks had a significantly lower adhesion rate due to their inability to bend along the robot’s skin as it moved on its crawler belt.

Regarding peeling, the use of water as the adhesive remover proved effective, with the highest peeling rates observed with paper and Color Zeolite. Paper peeled off more easily as multiple sheets came off together when one was removed. However, Color Zeolite adhered more strongly due to its granular nature, requiring external force to peel it off. Adding external force improved the peeling rate.

The implementation of the liquid extrusion mechanism showed that the robot could detach its exterior without relying on external devices. However, the mechanism provided uneven liquid distribution, indicating the need for more uniform liquid supply mechanisms.

## 5. Conclusions

In this study, we described the robot that acquires external information from the environment and the experiments that were carried out. The results demonstrated that the water-soluble adhesive has sufficient adhesive strength to capture external materials and that these materials can be removed using water. In our previous research, the only material that could be bonded was small sand. In contrast, in this study, the robot’s skin and adhesives were improved, enabling it to collect larger and heavier materials than pieces of wood, branches, and leaves.

However, some issues were identified. External materials, such as small twigs, did not adhere well to the robot’s skin, and the peeling rate was low, proving insufficient. In the case of natural materials, it is thought that this may be because their shapes are uneven, making it difficult for the adhesive to adhere stably. Additionally, the liquid extrusion mechanism has several challenges, such as a small amount of liquid being dispensed from the pouch and an uneven distribution of the liquid when extruded. The uneven distribution of liquid is likely because the outlet of the liquid extrusion mechanism faces forward, which tends to push the liquid toward the center of the robot.

The possible applications we are considering are as follows:

1. Camouflage from the surrounding environment.
2. Strengthening the exterior of the robot itself.

Because the robot can collect light materials such as sand and leaves, it is believed that camouflage is close to being realized to a certain extent. On the other hand, the robots developed so far have not yet been able to gather enough external materials, so further research is needed to strengthen the exterior.

In the future, based on the results of the adhesion and peeling experiments, efforts will be made to improve both the adhesion rate and the peeling rate of external materials. Currently, the liquid is extruded from a triple-hole dispenser, but a more uniform release of liquid onto the skin is necessary. Moreover, the addition of devices that assist in adhesion and peeling, such as a mechanism to press the skin against the ground, is crucial. Also, developing a mechanism capable of supplying more liquid and examining the robot’s shape and external environment to ensure more uniform adhesion of the exterior to the robot are important steps. It is necessary to develop a mechanism to prevent clogging and leakage during long-term use. It will also be necessary to carry out durability tests to verify its performance.

Considering future implementation, it will be important to investigate the degree of deviation in the robot’s collection ability when performing the same action on a specific object. Hence, we would like to conduct further experiments and investigate the stability of the robot’s collection ability. In the current trials, we measured the basic performance of the robot on flat ground to reduce external factors and evaluate its basic performance. However, for practical use, it will be important to confirm its performance in dynamic or complex terrain.

We also want to consider a mathematical model that can theoretically examine the model we propose in the future. At this point in time, we have not yet found a concrete idea of which parts the mathematical model should be applied to. However, it would be meaningful to find such a model in the future. These improvements will aim to enable the robot to enhance itself by acquiring external materials, ultimately achieving the adhesion and peeling of larger and more numerous external substances.

## Figures and Tables

**Table 1:** Adhesive requirements and comparison.

	1	2	3
Water	No	Yes	Yes
Instant adhesives	Yes	No	No
Liquid adhesive	Yes	Yes	No
Water-soluble adhesive	Yes	Yes	Yes

## Data Availability

Data are contained within the article.
